# Effect of Bloodletting at Shaoshang and Shangyang Acupuncture Points on Outcome and Prognosis of Severe Community-Acquired Pneumonia in the Elderly

**DOI:** 10.1155/2021/3295021

**Published:** 2021-10-26

**Authors:** Yuefeng Fu, Zhe Yang, Yangping Cai, Hongshuan Liu, Shuo Li, Nan Kou, Jingqin Wu, Qing Zhang

**Affiliations:** ^1^Emergency Department, DongCheng District Branch, Dongzhimen Hospital Affiliated to Beijing University of Chinese Medicine, BeiJing 100700, China; ^2^Department of Intensive Care Unit, Tongzhou District Branch, Dongzhimen Hospital Affiliated to Beijing University of Chinese Medicine, Beijing 101100, China

## Abstract

**Objectives:**

The aim of this study was to explore, whether treatment with bloodletting at Shaoshang and Shangyang acupuncture points would affect therapy outcome and prognosis for severe community-acquired pneumonia (SCAP) in the elderly.

**Methods:**

A total of 62 patients, who met the diagnostic criteria for SCAP, were enrolled in the study and randomly divided into two groups, i.e., treatment group (*n* = 31) and control group (*n* = 31). All patients received a therapy according to the Chinese Clinical Practice and Expert Consensus of Emergency Severe Pneumonia from 2016. In addition to that, a bloodletting at Shaoshang (LU11) and Shangyang (LI1) acupuncture points was applied for the treatment group. This intervention was repeated for three times (ones daily), bloodletting a volume of 2-3 ml at each time point. Differences in a main index of clinical efficacy, body temperature (T), respiratory rate (RR), heart rate (Hr), white blood cell count (WBC), neutrophil percentage (N%), and C-reactive protein level (CRP) as well as different scores (CURB-65 score, SOFA score, and Apache II score) were compared between groups. Moreover, the 28-day mortality was compared between treatment and control group. The statistical methods involved in carrying out the current study include *t*-test, Wilcoxon test, and chi-square test.

**Results:**

The clinical effective rate of the treatment group was 82.9%, which was significantly higher than the 17.1% in the control group (*P* < 0.05). After finishing the intervention, the treatment group showed significantly lower T (37.28 ± 0.54 vs. 37.82 ± 0.81), RR (20.06 ± 2.67 vs. 23.71 ± 6.85), Hr (81.71 ± 10.38 vs. 93.84 ± 15.39), CUBR-65 score (2.16 ± 0.74 vs. 3.03 ± 0.98), and SOFA score (5.84 ± 3.83 vs. 8.16 ± 4.2) compared to the control group (*P* < 0.05). The 28-day mortality rate of the treatment group was significantly lower than in the control group (12.9% vs. 45.2%, *P* = 0.05).

**Conclusions:**

Bloodletting at Shaoshang and Shangyang acupuncture points can support improving the clinical treatment efficacy for SCAP and reduce the 28-day mortality rate in the elderly.

## 1. Introduction

Related to a growth in proportion of the elderly population, the number of patients with community-acquired pneumonia (CAP) also increases. CAP is a common disease that occurs mostly in the elderly. It usually causes severe community-acquired pneumonia (SCAP) and may be associated with increased mortality [1]. Among elderly CAP patients, 75% of patients require hospitalization, and up to 10% of them develop SCAP and need to be admitted to the intensive care unit (ICU) [2]. During SCAP, critically ill patients often require mechanical ventilation, which is a dangerous situation that may be related to myocardial injury or infarction and acute heart failure. SCAP ranks as the sixth cause of death in the United States. The mortality rate of patients admitted to the ICU is about 40%, while the current reported mortality rate in China is about 53% [3].

Various clinical symptoms of SCAP include systemic inflammatory response syndrome (SIRS) containing temperature (more than 38°C or less than 36°C), breath (more than 20 times per minute), HR (more than 90 times per minute), white blood cells (more than the level of 12 × 10^9^/L or less than 4 × 10^9^/L), or neutrophilic granulocyte band form (more than ten percentage). Traditional Chinese medicine (TCM) believes that SIRS is related to syndrome of heat toxin inside. This is based on the abovementioned clinical manifestations. There are many scales that contain the use of CRUB-65, SOFA, and Apache II to assess the severity of the condition, and as we know the score is higher, the condition is more serious. The CRUB-65 which is used frequently provides information on the statement of severity of disease [4], when this score is 3 or higher, the condition of patients is considered as gravely illness. The CRUB-65 contains five elements, including confusion changing (C), urea > 7 mmol/L (U), respiratory rate > 30 (R), systolic blood pressure < 90 mmHg or diastolic blood pressure < 60 mmHg, and age > 65 years. For each of these issues, one point is added to the scale.

In short, the treatment of SCAP is difficult, and the prognosis is often worse, especially in the elderly. SCAP is an infectious disease, and antibiotics are the main drugs to treat the disease. Streptococcus pneumoniae and atypical pathogens are the main pathogens of SCAP [5], but with the use of broad-spectrum antibiotics, microorganisms are very likely to develop drug resistance during the treatment process, and multiple drug-resistant bacteria are often detected [6], which leads to a prolonged course of disease and ultimately an increase in mortality. Therefore, a supportive treatment plan is needed to improve the outcome and prognosis of SCAP. Acupoint bloodletting therapy in traditional Chinese medicine has a long history and has the clinical effect of lowering body temperature to treat fever.

SCAP belongs to the category of wind-warming lung-heat, which is usually treated by clearing lung heat away in TCM. The lung and large intestine contact outside and inside with each other, so unblocking the fu-organs can help eliminate lung heat. Shaoshang (LU 11) acupuncture point belongs to the Taiyin lung meridian of the hand, and the Shangyang (LI 1) acupuncture point belongs to the Yangming large intestine meridian of the hand. It is said that bloodletting at these two acupuncture points has the clinical effect of clearing lung heat and dredging the fu-organs [7, 8], while it is unclear, whether these bloodletting acupuncture points could be supportive in treatment of SCAP. Therefore, we developed this study to examine whether regular treatment with bloodletting at Shaoshang and Shangyang acupuncture points would affect therapy outcome and prognosis more effectively than conventional treatment for SCAP in the elderly. To achieve this research goal, the use of prospective randomized controlled trials was employed by exploring and examining clinical efficacy, 28-day mortality rate, and scores of critically ill patients.

## 2. Patients and Methods

### 2.1. Study Design

This investigation was designed as a randomized, controlled clinical trial. The study protocol has been approved by the Ethics Committee of Dongzhimen Hospital Affiliated to Beijing University of Chinese Medicine (approval no. DZMEC-KY-2019-146) and registered at the International Center for Clinical Trials (registration no. ChiCTR2100049339). All patients gave their informed consent for participation.

### 2.2. Randomization

A total of 62 random numbers were entered in column A1 of an Excel sheet. Then, the formula = RAND() was pasted in B1. Subsequently, “Enter” was clicked; then, data were dropped down, and generated data were sorted in ascending order. Afterward, the first 31 data were combined into treatment group and the last 31 number into control group. The patients were assigned to either treatment group or control group, respectively. This process ensures that each eligible patient was assigned to any group with the same probability.

### 2.3. Diagnostic Criteria of Severe Community Acquired Pneumonia (SCAP)

All participants were patients in the intensive care unit, Dongzhimen Hospital, Beijing University of Chinese Medicine between January 2019 and May 2021. This study employed the diagnostic criteria from the Clinical Management Guidelines for community-acquired pneumonia (CAP). The diagnostic criterion of CAP contains the following:

1. Community-acquired disease
2. The symptoms of pneumonia include (a) getting cough and sputum recently or exacerbation of the original respiratory disease symptoms, with/without purulent sputum/chest pain/dyspnea/hemoptysis; (b) fever; (c) lung consolidation and/or hearing wet rale; and (d) white blood cell count higher than 10 × 10^9^/L or less than 4 × 10^9^/L, with/without left shifting of nuclei
3. The chest X-ray showed newly appeared patchy infiltrates, leaf/segment consolidation, ground glass shadows, or interstitial changes, with/without pleural effusion

Patients who met (1) and (3) and any one criterion in (2) were diagnosed with CAP. The CRUB-65 was used to evaluate the severity of CAP. In case of a score of 3 or higher, the condition of patients was interpreted as severe. This score contains five elements, i.e., confusion changing (C), urea > 7 mmol/L (U), respiratory rate (RR) > 30 bpm, systolic blood pressure < 90 mmHg or diastolic blood pressure < 60 mmHg, and age > 65 years. Patients who fulfilled the criteria for CAP and had a CURB-65 score of 3 or more were considered as SCAP and included in the current study.

### 2.4. Patient Selection Criterion

The following inclusion criteria were defined:

- Patients having SCAP, who were hospitalized in the intensive care unit of Dongzhimen Hospital of Beijing University of Chinese Medicine from 2019 to 2021
- Body temperature > 38.5°C
- Age > 60 years (calculated based on date of birth)
- Patients or immediate family members provided written informed consent

Patients with the following diseases or conditions were excluded from this trial:

- The investigator judged that the patients could not complete therapy or be unsuitable to participate in the study because they might die within 48 hours, while they refused to accept active treatment
- The thumb or index finger was absent
- Patients with severe hospital-acquired pneumonia
- Patients with ventilator-related severe pneumonia
- Patients with other infections beyond pulmonary infections, such as abdominal cavity infections, urinary tract infections, or intracranial infections
- Patients with severe coagulation dysfunction
- Patients under hormones or immunosuppressants or other drugs for a long term
- Patients with malignant tumors, liver cirrhosis, chronic renal failure, blood system diseases, immune system diseases, acquired immune system diseases (HIV), or other serious basic diseases
- Patients with pregnancy or breastfeeding
- Patients participating in other clinical trials

Patients with the following situation were removed from the experiment:

- Patients that failed to follow-up with treatment according to the study protocol more than 24 hours after enrollment
- Patients who started using special treatment drugs (hormones, immunosuppressive agents, and traditional Chinese medicine injections that could clear heat away or resolve phlegm like *Tanreqing Injection*, *Reduning Injection*, *Qingkailing Injection*, etc.) after they were enrolled for the study were removed from the experiment since such drugs could affect the evaluation of the efficacy

Patients with the following condition were discontinued and withdrawn:

- Due to complications, other treatment measures were required, and the patients were unfit to continue the experiment
- The subject was pregnant, during the study period
- Subjects or family members requested to withdraw from the study

### 2.5. Intervention

All included patients received the therapy according to the Chinese Clinical Practice and Expert Consensus of Emergency Severe Pneumonia from 2016 [9]. In addition, the treatment group underwent three days (once daily), bloodletting a volume of 2-3 ml each time point. The bloodletting treatment process was done as follows: the first thing was to press the acupuncture site up and down with the thumb and the index finger to initiate the accumulation of blood on the site, then using 0.5%-1% iodine volt to disinfect the fingers. After wearing sterile gloves, the left thumb, the index finger, and the middle fingers are used to pinch the acupuncture site. The needle handle is held with the right thumb and the corresponding index finger; meanwhile, the right middle finger pulp is close to the lower end of the needle body. The exposed length and pierced depth of the needle tip were 3-5 mm at two disinfected acupuncture sites. After puncture, a 2-3 ml blood volume was drained (approximately 2 sterile cotton balls), and in the case in which the bleeding was not smooth, it was squeezed out. After completing the bloodletting, the needle hole was pressed with sterile cotton balls for one minute until stanched. Acupuncture points included Shaoshang (LU 11) and Shangyang (LI 1) acupoints.

The positioning of the bloodletting points was determined concerning the national standard of the People's Republic of China, “Name and Position of Acupoints” (GB/T 12346-2006) [10]. Shaoshang (LU 11) is located on the radial side of the finger, at the end of the thumb, 0.1 inch above the corner of the nail base. Shangyang (LI 1) is located on the radial side of the distal segment of the index finger, 0.1 inch away from the nail angle.

### 2.6. Observation Indices

#### 2.6.1. Main Efficacy Index

To assess the efficacy of the measures, a specific index was applied. After finishing the treatment, it was considered effectively, when the patients met all of the following criteria: (I) the body temperature (T) ≤ 37.2°C, heart rate (Hr) ≤ 100 beats/min (bpm), respiratory rate (RR) ≤ 24 breaths/min (bpm), systolic blood pressure (SBP) ≥ 90 mmHg, blood oxygen saturation (SP0_2_) ≥ 90% (or PaO2 ≥ 60 mmHg), and white blood cell count (WBC) within the reference range.

#### 2.6.2. Secondary Efficacy Indices

T, WBC, neutrophil percentage (N%), C-reactive protein level (CRP), CURB-65 score, SOFA score, and Apache II score had been recorded at the day before enrollment. The T, WBC, N%, and CRP had been recorded every day after enrollment until the end of intervention. The index of CURB-65 scores, SOFA score, and Apache II score had been recorded before and after finishing treatment. Survival status was determined by follow-up until 28 days after enrollment. All the indices were used to evaluate the outcome in the respective groups.

### 2.7. Statistical Analysis

The statistical software (SPSS version 24.0) was used for statistical analysis. The normal-distributed measurement data were tested by paired *t*-test, and the results were expressed as mean ± standard deviation. Wilcoxon test was used for measurement data that did not show normal distribution or showing uneven variance, whereby the results are expressed by the median and interquartile range. The count data were tested by chi-square test (*X*^2^) and expressed by percentage. The difference was considered as statistically significant at *P* value < 0.05.

## 3. Results

### 3.1. Baseline Characteristics of Patients

A cohort of 62 patients was enrolled in the study, of which all completed the follow-up. 31 individuals were in the treatment group (TG), and 31 were in the control group (CG). Clinical characteristics and comparisons between 62 patients are shown in Table 1. In TG, 22 males and 9 females, aged from 62 to 82 years (70.58 ± 6.49), and with a body mass index of 24.03 ± 3.15 were examined. In CG, there were 11 males and 20 females, aged 61-88 years old (73.74 ± 8.21) years and with a body mass index of 21.13 ± 2.51. No significant differences were found between groups (*P* > 0.05), indicating that the groups were comparable.

### 3.2. Comparison of Clinical Treatment Efficacy and 28-Day Mortality

The levels of T, RR, and Hr were significantly lower in TG compared to CG after finishing treatment (*P* < 0.05). At baseline, the level of PO_2_ in TG was lower than in CG (*P* < 0.05), but after finishing treatment, the level of PO_2_ was significantly higher in TG compared to CG, as shown in Table 2. Comparing the differences in efficacy between the groups, better results were found in TG compared to CG (*P* < 0.05). After finishing treatment, the effective rate of TG was 82.9%, and of CG, it was 17.1%. The TG had lower 28-day mortality compared with CG (12.9% vs. 45.2%; *P* = 0.05, Table 3).

### 3.3. Comparison of Infection Index

**Figure 1:**
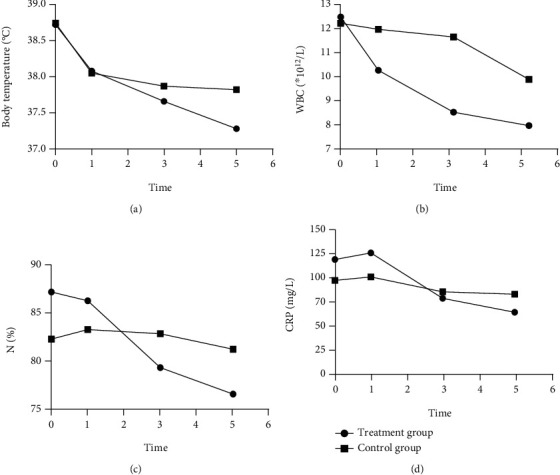
Comparison of infection indicators between treatment group and control group. Data were expressed as mean ± s (*n* = 31). (a) Body temperature changes between two groups during treatment (*P* < 0.05). (b) White blood cell count changes between two groups during treatment (*P* > 0.05). (c) Percentage of neutrophils changes between two groups during treatment (*P* > 0.05). (d) C-reactive protein changes between two groups during treatment (*P* > 0.05).

Infection Index contained T, WBC, N%, and CRP. There were no significant differences in these indices at baseline between two groups (*P* > 0.05). After treatment, there were no significant differences in WBC, N%, and CRP between two groups (*P* > 0.05), but the level of these indices were lower in TG, and the level of T was significantly lower in TG compared to CG (*P* < 0.05), as shown in Figure 1.

### 3.4. Comparison of Score

There were no significant differences in CUBR-65 score, Apache II score, and SOFA score at baseline between two groups (*P* > 0.05), while significant differences between TG and CG were revealed in CUBR-65, Apache II and SOFA score after treatment (*P* < 0.05). The results are shown in Table 4.

## 4. Discussion

As far as we know, this is the first bloodletting report combining Shaoshang acupuncture points with Shangyang to treat SCAP based on the basic theory of TCM. SCAP is caused by a microbial infection that leads to an inflammatory response or SIRS (described in the introduction). Temperature, heart rate, respiratory rate, and WBC levels are used to assess the severity of inflammation, and the WBC level, N%, and CRP are considered the first indices to assess the severity of inflammation. Therefore, we selected these indices in our study to evaluate the inflammation of effectiveness. We found that temperature, HR, and RR in TG were more effective in the decrease than in CG, and the values of WBC, N%, and CRP were lower after treatment than in CG, which suggests a better efficacy of bloodletting in combination with the regular therapy in the old population with SCAP. Meanwhile, temperature, heart rate, respiratory rate, and WBC levels were the important indices to assess the effectiveness of treatment for SCAP, and the desirable results of this study were the clinical effectiveness of SCAP therapy compared to regular basic therapy in the elderly.

Severe infections can lead to sepsis and even multiple organ dysfunction. Therefore, monitoring the severity of the disease and the function of internal organs is also important in the treatment process. CRUB-65 is often used to assess the severity of CAP, and SOFA is often used to monitor the degree of organ damage. APACHE II is currently the most widely used and authoritative critical illness assessment system, which can be used as an index to evaluate the condition and prognosis of ICU patients. Therefore, we selected these indicators to evaluate their effectiveness. The results showed that the scores of CRUB-65, SOFA, and APACHE II in the TG group were significantly lower than those in the CG group, indicating that the TC group had better internal organ's function protection.

The 28-day mortality rate is another commonly used indicator in clinical research. In this study, in TG, it was 12.9% compared with 45.2% in CG, and the statistical value result was 0.05. The statistical value might be significantly different if the sample size could be expanded.

SCAP is a common critical illness in the elderly that presents with fever, cough, expectoration, dyspnea, and increased level of WBC, N%, and CRP, and finally leading to multiple organ dysfunction syndrome (MODS). Clinically, elderly individuals suffering from SCAP could have a relatively higher potential of a life-threatening course, whereby their mortality rate is reported to be around 48% [11]. Since SCAP can significantly negatively affect quality of life and is a leading cause of mortality in the elderly. In this study, the mortality rate was 12.9% in TG compared to 45.2% in CG. Bloodletting at the Shaoshang and Shangyang acupuncture points may be an effective therapy measure for SCAP in the elderly.

Fever is a special symptom of SCAP, when the body temperature increases 1°C, the basal metabolic rate will increase by about 10%, potentially causing abnormal metabolism of the three major nutrients. When the body temperature is higher than 40°C, the blood temperature will affect the respiratory control center and increases its sensitivity to carbon dioxide, this leads to short breath, and elderly patients are more likely to have disturbance of consciousness with wheezing, which seriously affects blood oxygen and requires invasive ventilator breathing support. In addition, elderly patients usually show a reduced cardiac reserve, what is often associated with coronary atherosclerosis. The increased body temperature and increased heart rate may lead to insufficient blood and oxygen supply to the heart, potentially causing myocardial injury, acute heart failure, or even acute myocardial infarction. At the same time, the reduced secretion of digestive juice can be induced by fever. Moreover, the activity of digestive enzymes will decrease, leading to gastrointestinal dysfunction. Therefore, it is also very important to lower the patient's body temperature timely, especially in the elderly. Compared to CG in our study, body temperature in TG was significantly lower after treatment. This suggests that bleeding at the two acupuncture points may play a protective role in the treatment of SCAP. Regardless, thanks to the basic theory of TCM for providing the guide to the choice of these two acupuncture points.

TCM has a long history of acupuncture and bloodletting therapy. Simultaneously, its clinical efficacy is remarkably based on the differentiation of symptoms and signs of meridians. TCM considers that SCAP belongs to wind-warming lung-heat and employing heat-clearing as a main treatment method [12–14]. Acupuncture point bloodletting therapy is derived from the “Inner Canon of Huangdi” and has the functions of dredging the meridians, promoting blood circulation, and clearing away heat and detoxification as well as dispelling evils [15, 16]. Thereby, it is the first choice to reduce fever according to ancient Chinese Medicine Book “Lingshu, Fever” [17]. Nowadays, because bloodletting at acupuncture points is easy to implement, it has been used widely by traditional Chinese medical practitioners.

On the other hand, TCM considers that Shaoshang acupuncture point is the “well-point of the lung” and the source of lung diseases, making it useful for the therapy of respiratory diseases. It is assumed that the evil influence of the lung meridian can be discharged through bloodletting. Many clinical studies have reported that bloodletting is used to treat upper respiratory tract infections and related diseases, especially the improved reduction of the body temperature in context of respiratory diseases has been highlighted [18–20]. Shangyang acupuncture point is the “well-point of the large intestine meridian” of Hand Yangming. The large intestine connects with the lung outside and inside. When the lung suffers from evil leading to dysfunction, the large intestine meridian will be involved, presenting bloating and constipation, which means symptom of intestinal block. Therefore, it is necessary to combine two acupuncture points with bloodletting to treat lung diseases. Studies have shown that two acupuncture points combined with bloodletting not only have the potential to lower the body temperature of patients with upper respiratory tract infection but also can relieve local swelling and pain [21]. Another study showed that two acupuncture points combined with bloodletting lead to a better clinical efficacy than the single-use antibacterial or antiviral drug application [22]. These issues are in line with the current study's findings and support the benefit of acupuncture point bloodletting as adjunctive treatment for SCAP.

This research has many advantages. First, bloodletting at acupoints is easy to implement, and patients without thumbs, index fingers, and coagulation disorders can all receive this therapy. Second, compared with conventional basic therapies, this treatment method helps support the clinical efficacy of SCAP therapy in the elderly. Third, bloodletting is 22 Renminbi per time in our hospital, which is cheaper than antibiotics. However, it is also worthwhile to mention the several limitations existed in the present study. This research was a single-centered, small sample-sized study. The absence of a power calculation needs thereby to be mentioned as limitation. The in- and exclusion criteria were very comprehensive, to ensure the exclusion of potential confounding factors. However, a certain heterogeneity of groups remains possible. Moreover, antibiotics play a key role in the treatment of SCAP, and the conventional therapeutic regimen may be diverse in different cases, especially when it comes to antibiotic selection. Furthermore, the magnitude of intervention was quite short (three days), and the observational period was restricted to about one month. Therefore, there is no statement on potential long-term effects possible at the moment. These problems call for a deeper strengthening of this research since we could only perform simple analysis on clinical efficacy evaluation. Although this is still a follow-up study, it still provides some preliminary support for the effectiveness of Shaoshang and Shangyang bloodletting in the treatment of SCAP. In addition, a large prospective study is intensively required to assess the effectiveness and safety of the bloodletting at Shaoshang and Shangyang acupuncture points in management of SCAP and to identify further potential mechanisms.

## 5. Conclusion

In conclusion, the results of this randomized controlled trial suggest that the two acupuncture points combined bloodletting therapy have better clinical efficacy on supporting SCAP-therapy than the conventional treatment alone in the elderly. Thereby, bloodletting had also an effect on reducing the 28-day mortality rate, making it recommendable as adjunctive therapy in elderly suffering from SCAP.

## Figures and Tables

**Table 1:** Comparison of baseline patient characteristics.

Group	Age (year)	Gender (male)	Body mass index
Treatment (*n* = 31)	70.58 ± 6.49^@^	22^#^	24.03 ± 3.15^$^
Control (*n* = 31)	73.74 ± 8.21	11	21.13 ± 2.51

^@#$^ *P* > 0.05, comparison to the control group at baseline.

**Table 2:** Comparison of effective index.

Variables	Time point	Treatment group (M ± SD)	Control group (M ± SD)	*t*	*P*
Hr (bpm)	Day 0	111.61 ± 23.66	104.87 ± 22.52	1.149	0.255
	Day 3	85.84 ± 14.14	95.52 ± 14.27	-2.635	0.011
	Day 5	81.71 ± 10.38@	93.84 ± 15.39@	-3.638	0.01#

RR (bpm)	Day 0	32.35 ± 5.56	33.61 ± 5.77	-0.874	0.386
	Day 3	21.32 ± 3.35	23.94 ± 7.22	-1.829	0.072
	Day 5	20.06 ± 2.67@	23.71 ± 6.85@	-2.76	0.008#

PO_2_ (mmHg)	Day 0	79.44 ± 29.10	100.04 ± 34.00	-2.564	0.013
	Day 3	112.23 ± 40.77	103 ± 35.90	0.928	0.357
	Day 5	101.90 ± 26.84@	95.52 ± 32.39	0.845	0.401

N%	Day 0	87.03 ± 8.11	82.41 ± 10.34	1.961	0.54
	Day 1	86.17 ± 7.82	83.34 ± 8.82	1.335	0.187
	Day 3	79.61 ± 9.20	82.93 ± 9.30	-1.418	0.161
	Day 5	77.00 ± 9.70@	81.40 ± 8.01	-1.959	0.055

CRP (mg/L)	Day 0	117.75 ± 83.31	96.76 ± 69.82	1.868	0.240
	Day 1	124.46 ± 84.79	100.35 ± 71.80	0.768	0.446
	Day 3	78.83 ± 60.06	85.3 ± 67.94	-3.97	0.693
	Day 5	64.82 ± 47.29@	82.90 ± 61.24	-1.3	0.189

T (°C)	Day 0	38.72 ± 0.28	38.74 ± 0.32	-0.21	0.835
	Day 1	38.08 ± 0.63	38.05 ± 0.85	0.136	0.892
	Day 3	37.66 ± 0.67	37.87 ± 0.96	-0.992	0.325
	Day 5	37.28 ± 0.54@	37.82 ± 0.81@	-3.08	0.03#

WBC (^∗^10^12^/L)	Day 0	12.49 ± 5.33	12.24 ± 6.31	0.17	0.866
	Day 1	10.37 ± 4.11	12.00 ± 7.26	-1.09	0.281
	Day 3	8.70 ± 4.42	11.70 ± 5.81	-2.3	0.026
	Day 5	8.16 ± 3.74@	10.00 ± 4.33	-1.8	0.084

@*P* < 0.05, compared to before treatment. #*P* < 0.05, compared to the control group after treatment.

**Table 3:** Comparison of efficacy and 28-day mortality between the two groups.

	Treatment group (*n* = 31)	Control group (*n* = 31)	*X* ^2^	*P*
28-day mortality	12.9%	45.2%	7.828	0.05
Effective rate	82.9%	17.1%	34.707	<0.01

**Table 4:** Comparison of scores between treatment group and control group.

Variables	Time point	Treatment group (*n* = 31)	Control group (*n* = 31)	*t*	*P*
CUBR-65	Day 0	3.45 ± 0.62	3.68 ± 0.54	1.52	0.133
	Day 3	2.52 ± 0.77	3.03 ± 1.05	-2.21	0.31
	Day 5	2.16 ± 0.74	3.03 ± 0.98	-3.962	0.01^∗^

Apache II	Day 0	20.71 ± 9.92	19.13 ± 7.61	0.74	0.484
	Day 3	14.65 ± 8.66	16.81 ± 7.86	-1.029	0.308
	Day 5	13.00 ± 6.62	17.77 ± 7.21	-2.785	0.007^∗^

SOFA	Day 0	7.19 ± 3.50	6.97 ± 2.93	0.277	0.783
	Day 3	6.06 ± 3.92	7.94 ± 4.08	-1.84	0.71
	Day 5	5.84 ± 3.83	8.16 ± 4.2	-2.274	0.027^∗^

^∗^ *P* < 0.05, comparing to the control group after treatment.

## Data Availability

The data that support the findings of this study are available on request from the corresponding author.
